# Engaging male students with mental health support: a qualitative focus group study

**DOI:** 10.1186/s12889-020-09269-1

**Published:** 2020-07-24

**Authors:** I. Sagar-Ouriaghli, J. S. L. Brown, V. Tailor, E. Godfrey

**Affiliations:** 1 Department of Psychology, Addiction Sciences Building, Institute of Psychiatry, Psychology & Neuroscience (IoPPN), King’s College London, 4 Windsor Walk, Denmark Hill, London, SE5 8BB UK; 2 Department of Psychology, Henry Wellcome Building, Institute of Psychiatry, Psychology and Neuroscience (IoPPN), King’s College London, London, SE5 8AF UK; 3 GKT School of Medical Education, King’s College London, London, UK; 4 Department of Population Health and Environmental Sciences, Faculty of Life Sciences and Medicine, King’s College London, London, SE1 9RT UK; 5 Psychology Department, Guy’s Campus, 5th Floor Bermondsey Wing, London, SE1 9RT UK

**Keywords:** Help-seeking, Men, Interventions, Students, Mental health

## Abstract

**Background:**

Males are less likely to seek help for mental health difficulties compared to females. Despite considerable interest, a paucity of evidence-based solutions exists to address this. Concerns about students’ mental health has led to the United Kingdom’s Department of Education to make this a priority. Studies have shown that male students hold more negative attitudes towards the use of psychological services compared to female students and are less likely to seek help. A major concern is that male students make up 69% of university suicides, which is often associated with lower rates of help-seeking. This focus group study therefore sought to identify potential approaches that would be relevant to improving mental health help-seeking in male students.

**Methods:**

Three focus groups comprising of 24 male students at a London University were conducted. Participants were asked questions exploring: the barriers to seeking help, what would encourage help-seeking, how an appropriate intervention should be designed, and how to publicise this intervention to male students. Thematic analysis was conducted to evaluate participants responses.

**Results:**

Five distinct themes were identified. These were: 1) protecting male vulnerability, 2) providing a masculine narrative of help-seeking, 3) differences over intervention format, 4) difficulty knowing when and how to seek help, and 5) strategies to sensitively engage male students.

**Conclusions:**

These themes represent important considerations that can be used, together with the existing literature about male help-seeking, to develop more male friendly interventions that are suitable for male students. This could help improve help-seeking attitudes and the uptake of mental health interventions for male students experiencing emotional distress.

## Background

The United Kingdom (UK) is increasing its efforts to tackle issues surrounding student mental health. The majority of students fall into the age bracket of 18–25 years, coinciding with the peak onset period for various mental health disorders such as schizophrenia, and anxiety and depression [1, 2]. The Department of Education is developing guidelines to ensure universities improve the mental health support offered to students [3, 4]. Such initiatives can be attributed to the rise in students reporting mental health conditions. From 2007 to 2017 five times as many students disclosed a mental health condition, reflecting a 12% increase across a 10-year period [5]. Problems such as anxiety and depression are common in university students [6]. Additional concerns of suicidal thoughts and behaviours, problematic drinking and substance misuse also occur frequently in this population [7–9]. Alongside the increase in students reporting common mental health problems, it has been noted that symptoms have become more severe [10]. This has increased the demand on student mental health services, which continues to rise annually [10, 11]. These factors, coupled with the stressors of university, can have a detrimental impact on academic performance [12] and place students at a greater risk of dropping out [13].

An additional problem is that students are often still reluctant to seek help for mental health difficulties [14]. The stigma associated with seeking help has been shown to reduce student’s willingness to talk about their mental health concerns [15]. Confidentiality, trust, poor symptom awareness, self-reliance, inadequate service knowledge and difficulty expressing emotions have also been highlighted [16, 17]. Further inspection of these barriers shows that they differ by gender. Indeed, female students hold more favourable attitudes towards help-seeking compared to males [18]. Traditional masculine gender roles of stoicism, invulnerability and self-reliance can reduce men’s willingness to seek support [19, 20]. In one study, male students preferred to deny weakness in order to uphold a stoic position and limit self-disclosure to remain autonomous; interestingly, they were more likely to engage in mental health support when help-seeking was characterised as a sign of strength [21].

Despite these findings, there remains a dearth of evidence-based solutions that aim to improve male student’s help-seeking. Indeed, this is a key target area for universities and mental health services, particularly since 93 (69%) of the 134 students committing suicide in 2015, were male [5]. However, in the right circumstances, men are willing to talk about their emotional and physical experiences, including depression [22–24] and qualitative work has helped provide a better understanding of poor utilisation of mental health services [25].

The current study sought to conduct a series of focus groups with male university students. The aim of this research was to highlight key features that might be incorporated into mental health initiatives to help encourage male students to seek help for mental health difficulties.

## Method

### Design

Focus groups were chosen to explore the narratives of male university students as they are an effective strategy for collecting health data and a promising method to research mental health in men [26]. This approach can enhance the discussion of personal issues by proving a supportive group environment, resulting in richer findings that might not be obtained from individual interviews [27, 28]. Focus groups can help capture collective group attitudes, norms and overall narratives and foster positive group dynamics and interactions [27]. Discussion of mental health services with men should be encouraged [29], particularly as group discussion may also provide interpersonal support and validation for men experiencing psychological distress [30]. Purposive sampling was adopted to recruit participants to the focus groups as there was deliberate choice of participants based on their gender (i.e. male) and level of education (i.e. students) [31, 32]. Similarly, heterogenous purposive sampling was utilised in the current investigation to select a broad spectrum of participants regarding their ethnicity, previously help-seeking behaviours, and degree faulty to resemble the student cohort as closely as possible [31]. Thematic analysis was chosen to examine the narratives by breaking down speech into smaller units of content [33], as it is a method that seeks to identify, analyse and report patterns (referred to as themes) within the data [34].

### Patient & Public Involvement (PPI)

To develop a relevant topic guide, this research was initially reviewed by an advisory team with experience of mental health problems who have been specially trained to advise on research proposals and documentation through the Young Person’s Mental Health Advisory Group (YPMHAG) which is a free, confidential service in England provided by the National Institute for Health Research Maudsley Biomedical Research Centre via King’s College London (KCL) (https://ypmhag.org/) [35]. One author (ISO) presented the current investigation to the YPMHAG before seeking feedback. The YPMHAG consisted of 9 young adults (3 male) with a mean age of 22 years. Seven were either current or former university students.

The YPMHAG recommended that the investigation emphasise that the focus groups were not a form of group therapy, and that participants were not required to discuss personal experiences and any responses would remain anonymous. The finalised focus group questions explored the barriers to help-seeking, how to encourage mental health help-seeking, how mental health initiatives should be designed and how to publicise them to male students. A comprehensive topic guide is in supplementary material 1.

### Procedure

**Fig. 1:**
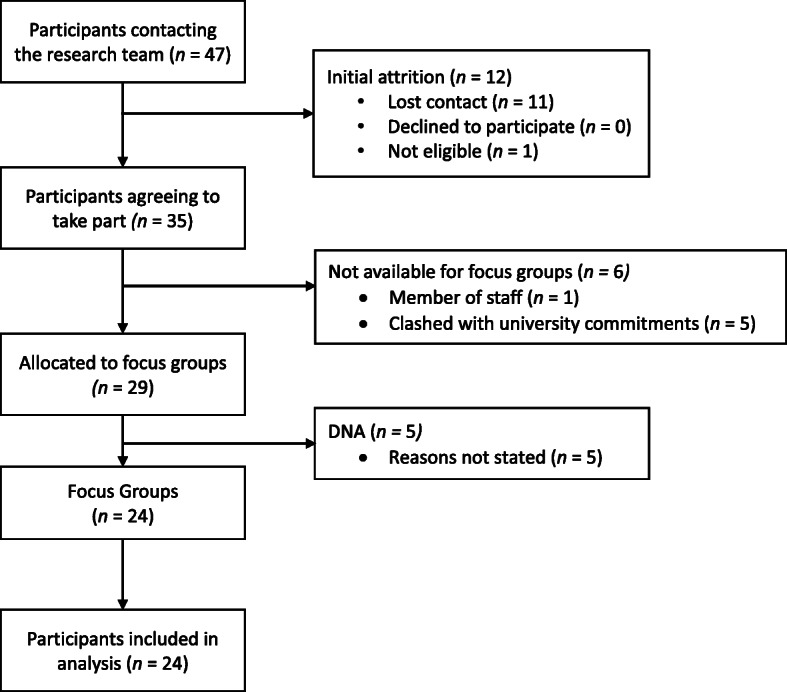
Recruitment flow chart

Ethical approval was granted by the universities local Research Ethics Office. The focus groups were advertised via a routine fortnightly e-mail used to recruit students to research studies that was sent to all students at the university. Posters were distributed across the university campus and posted within social media pages and various societies. Both the e-mail and posters contained a brief summary of the project and provided additional contact details if students were interested in participating. No prior relationships were established with potential participants before the study commenced. After contacting the research team, participants were sent an e-copy of the information sheet outlining the study in more detail including the aims of the research study and that it would be part of a PhD project. After reading this and agreeing to take part, participants were enrolled into the study. (Fig. 1). Upon arrival at the focus groups, situated in a university room located above the student’s union, participants were given a hard copy of the information sheet, provided with an opportunity to ask any further questions and completed a consent form to take part. Focus groups were conducted until data saturation, the point at which no new themes or concepts relating to the research question are interpreted from the data, was achieved [36–40]. Employing a thematic analysis approach, the research cannot determine exactly how many focus groups will be required in advance of analysis [40]. Transcripts of each focus group were reviewed after each session before conducting the next to determine if new concepts relating to the research question were identified within the data. Data saturation was reached after the third focus group whereby no new codes were developed. This is consistent with previous work as data saturation seeking to identify core themes within the data can be achieved with small sample sizes [37], with 84% of all possible codes being developed by the second focus group [37]. Additionally, 96% of high-prevalence codes can be identified by the third focus group [37]. Previous qualitative work investigating health-seeking behaviours of African American men found that two to three focus groups were effective for identifying 80% of all themes, and that three focus groups are enough to identify all of the most prevalent themes within the data set [40]. Three focus groups were facilitated by the lead researcher (ISO, PhD student, male), with the assistance of a medical student currently enrolled at the university (VT, male). A topic guide (supplementary material 1) was used to steer the conversation, but otherwise the facilitator allowed general discussion among the participants. During the focus groups, the second researcher took notes on the focus groups as well as the names of participants who had spoken and in which order to aid transcription. Other than the researchers and participants, no others were present.

### Data analysis

All focus groups audio discussions were recorded and encrypted on a Phillips Dictaphone Pocket Memo and transcribed verbatim by one author (ISO).

The six-step guide for Thematic Analysis recommended by Braun & Clarke [34] was used to analyse the data. The six steps are: 1) familiarisation with the data, 2) generate initial codes, 3) search for themes, 4) review themes, 5) define and name the themes, and lastly 6) produce the report. Coding was an iterative process conducted by two members of the research study team (ISO and VT) in an independent-parallel fashion before agreeing on finalised codes. Once all the data had been initially coded, it was categorised into broader groups encompassing relevant codes, which were abstract ideas expressed within the transcript and were agreed upon before identifying themes. Themes encapsulated a common phenomenon that emerges from reoccurring codes within the data and represented the most prominent ideas and experiences of the participants [41].

## Results

### Participants

Twenty-four male students attended the focus groups (Fig. 1) and were compensated for their time with a £20 Amazon voucher. Participants’ demographic information is outlined in Table 1. The mean duration of the three focus groups was 72.47 min.

**Table 1:** Participants’ demographic information

Demographics	***N*** (%)
Total number of participants (% male)	24 (100%)
Age (Years)	
Mean (SD)	21.89 (3.39)
Range	18–31
Ethnicity	
Chinese	7 (29%)
Any other white background	5 (21%)
White British	4 (17%)
Pakistani	3 (13%)
Black African/Caribbean	2 (8%)
Any other Asian background	2 (8%)
Arab	1 (4%)
Degree Faculty	
Institute of Psychiatry, Psychology & Neuroscience	5 (21%)
Natural & Mathematical Sciences	4 (17%)
Life Sciences & Medicine	4 (17%)
Business School	3 (13%)
Arts & Humanities	3 (13%)
Social Science & Public Policy	2 (8%)
Other/NA	2 (8%)
Dental Institute	1 (4%)
Level of Study	
Undergraduate	16 (67%)
Postgraduate (Master’s or PhD)	7 (29%)
Other	1 (4%)
Has previously sought help for mental health	
Yes	12 (50%)
No	10 (42%)
Prefer not to say	2 (8%)

**Fig. 2:**
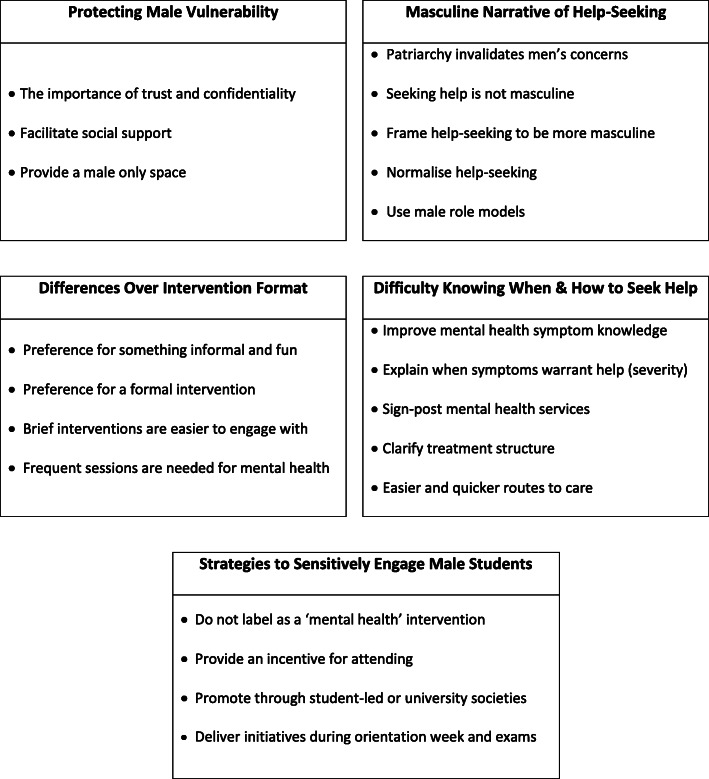
Overview of themes and sub-themes to improve mental health help-seeking for male students

Five distinct themes were identified. These were: 1) protecting male vulnerability, 2) providing a masculine narrative of help-seeking, 3) differences over intervention format, 4) difficulty knowing when and how to seek help and 5) strategies to sensitively engage male students. These results and their underlying sub-themes are summarised in Fig. 2.

### Theme 1: protecting male vulnerability

A prominent theme was that speaking about mental health was very difficult. The majority of participants were reluctant to confide in others and talk about their difficulties due to fears associated with opening up.

*“why would you want to open a can of worms? there is no point to that… not immediately anyway” – Participant 5, Group 3.*

*“most people just don’t know, most people just, they’re so afraid of what they don’t know they just like don’t want to know [talking about mental health]” – Participant 5, Group 1.*

To combat this, participants described environments that were safe and less threatening. They preferred settings that were more sensitive to male needs, enabling better management of the fear and vulnerability associated with opening up/seeking help. Many of the participants stressed the need for a safe space, trust and confidentiality.

*“they have to trust you because men aren’t like women, we don’t open up very easily we don’t” – Participant 5, Group 1.*

*“people need more information about confidentiality because a lot of people are afraid that if they say anything about their mental health problems, other people will find out and they may have problems with that” – Participant 9, Group 2.*

Others emphasised that talking about mental health with professionals can be a deterrent. Many had a preference for speaking to someone they knew such as a close friend or someone they have been briefly introduced to. Indeed, according to participants, this may help facilitate a trusting environment.

*“for me, it would be better if I will be surrounded by people who at least I know for like 10-15 minutes rather than a complete stranger – [if you say] ‘let’s talk about depression, or let’s talk about anxiety’. [I’ll say] not really, I don’t want to talk about it, I don’t know you guys why should I open up” – Participant 1, Group 1.*

Similarly, the importance of social support for psychological well-being and how this can encourage help-seeking was stressed.

*“maybe like a time like hanging out with a friend, socialise, but at the same time like seeking help” – Participant 1, Group 1.*

*“I think in my case a big help of this phase is actually people around me. So, like when I first experienced the issue, I didn’t seek help personally, it was the people around me” – Participant 1, Group 3.*

Furthermore, participants stated that a male-only space would also assist with protecting the vulnerability they experience when trying to seek help for mental health. Moreover, this could assist with validating difficulties male students may experience.

*“there’s so much available for literally everything else. Men are like, they’re pushed to one side, you don’t need the help as much as women, young children, older people, disabled people, but men, we have nothing for ourselves” – Participant 5, Group 1.*

*“I think emphasising men’s mental health is insanely important” – Participant 1 Group 3.*

*“I think if there were women here, I think it would detract from people like actually being open” – Participant 4, Group 3.*

These discourses emphasise the importance of providing a male only space or setting in which male students feel comfortable to disclose mental health concerns, whilst also providing an environment to facilitate further discussion around help-seeking. This may be enhanced by the assurance of trusting and confidential settings and facilitating social support with other male students.

### Theme 2: provide a masculine narrative of help-seeking

Traditional masculine stereotypes of being strong, responsible, invulnerable and self-sufficient were identified as key barriers to seeking help. Male students preferred to do things by themselves as seeking help contradicted masculine ideals.

*“responsibility that surrounds the male character is playing a huge role in this as well because if you are male and you have a lot of responsibility and then you know that, ‘okay I have a problem, now I have to seek help’, then you have to rely on someone else, then my responsibility is sort of, it could be that, I can’t do it anymore” – Participant 10, Group 1.*

*“you might also feel anxious about talking to people and then showing vulnerability, which is also a big part why guys just don’t talk about their emotions generally. They don’t want to show vulnerability” – Participant 2, Group 3.*

These points emphasise that seeking help reduces one’s ability to fulfil masculine ideals, particularly of responsibility and invulnerability. Furthermore, because men are often regarded as privileged in society they are not supposed to be disadvantaged. This in turn, makes it more difficult to open up about not feeling well or experiencing adversity.

*“the term patriarchy because it just infers that, that it’s impossible, or at least very difficult for men to have it bad, or to be disadvantaged in some way” – Participant 4, Group 3.*

Furthermore, the participants suggested that help-seeking appeared to be evaluated as an overall net loss. In this instance, seeking help would result in a loss to one’s masculine identity without necessarily any immediate benefit.

*“men especially, it’s [i.e. mental health] is always going to rank in the lower things you know, you’re never going to go, even like with regular health. I’m like ‘oh I think it’s broken but I’m not going to seek help immediately’” – Participant 5, Group 3.*

*“you have to make like a big commitment [to therapy], and this commitment is like a short-term loss, it’s a short-term loss”* – *Participant 1, Group 3.*

Concerns about responsibility, vulnerability and patriarchy infers that male students may benefit from a narrative that highlights how help-seeking can be masculine, will not be detrimental to their masculinity and engaging would be an overall net-gain. This was evidenced by some participants stating that help-seeking does not have to be weak and can be a sign of strength whilst working towards better health and personal growth.

*“if you tend to run away from your problems then you’re weak in this sense, not in the eyes of others, but towards yourself” – Participant 5, Group 2.*

*“people who attend then feel empowered because they’re doing something strong not weak. I’m here looking after myself and that’s empowering. It makes people who attend feel good and so I think that’s a really really good idea” – Participant 7, Group 2.*

One way to encourage help-seeking was to normalise the behaviour by emphasising that it was common. In addition, utilising male role models to talk about their own mental health experiences and help-seeking stories would inspire hope and reduce the perceived negatives associated with help-seeking.

*“I think just give them some materials or some something to the public that gives the feeling that seeking mental health [help] is not very special or a serious thing, just a normal thing, that it’s fine. So, when you just get a mental health problem you will feel easy to seek help” – Participant 3, Group 3.*

*“if they see another gentleman, high profession, high functioning individual, and they’re talking about XYZ, they might think ‘you know what, he’s done it, why not myself?’. If you could do a personal narrative that’ll be amazing” – Participant 5, Group 1.*

Overall, this theme highlights that help-seeking was perceived as a net loss to one’s masculine identity and male-students could feel disqualified from seeking support due to male-privilege. Indeed, framing help-seeking to fit masculine norms, as a normal act of self-care was suggested to improve male-students engagement with mental health interventions.

### Theme 3: differences over intervention format

Theme 3 highlighted a lack of consensus regarding the format of appropriate interventions for male students. These views were polarised, with participants disagreeing over the formality and duration of the intervention. Much of the discourse emphasised the need for a fun and informal structure to help promote engagement.

*“approach this from a different angle because we always do workshops, we always do lectures, we always do something which is like really formal rather than informal” – Participant 1, Group 1.*

*“something that’s fun, even if you are okay, something that you just come to anyway because it’s enjoyable, I definitely think that will be better” – Participant 7, Group 2.*

Equally, many participants felt the opposite and stated that they would prefer a formal and serious structure. This disagreement was centred around these participants perceiving mental health as serious and they were concerned that an informal group would not be structured enough to facilitate openness.

*“some people may be more open to sharing things if it’s in a more private setting. It may not be best to do it with a group of friends or anything like that” – Participant 4, Group 1.*

*“you don’t want to alienate people by making it seem so light-hearted, because it’s not. Because other issues are absolutely serious” – Participant 5, Group 1.*

The second disagreement was in response to the duration of the intervention. There was a preference, among half the participants, for something brief that lasted 1–2 h and was spread across one or two sessions.

*“an hour is fine, no-one has more than that to give away really” – Participant 6, Group 1.*

*“I can maybe come once but not more often, so there should be a tactic to reach people in one workshop” – Participant 2, Group 3.*

Conversely, others felt that multiple sessions that were repeated more frequently were a better format. This was due to a perception of mental health as a more enduring problem, thus requiring repetition of information and longer-term support to encourage help-seeking.

*“I know, even getting information, even getting information one session is not enough, you need repetition to get mental health across” – Participant 1, Group 3.*

*“I think one off things don’t actually work that much*” *- Participant 5, Group 2.*

Theme three captures the lack of consensus over the formality and duration of an intervention. This presents some difficulties when designing future mental health initiatives, but none-the-less demonstrates that these are salient factors, which may contribute to engaging male students with mental health support and other well-being practices.

### Theme 4: difficulty knowing when and how to seek help

Theme 4 provides an overview of how male students conceptualised mental health and determined appropriate action. Many students acknowledged their limited understanding of common mental health conditions, such as anxiety and depression, and how they present in men. Participants felt common mental health conditions and how they present should be addressed more openly to facilitate greater help-seeking. This should be explained in lay terms, as opposed to using medical terms, such as those from Diagnostic and Statistics Manual of Mental Disorders [42] and the International Classification of Diseases [43].

*“ask someone what depression means to you, and he’ll be like ‘err just someone who’s really sad’. Which is not necessarily clear, what we mean by it is that there’s biological changes, so they don’t understand it’s a lack of understanding and awareness”- Participant 5, Group 1.*

*“I think not necessarily describing it as a kind of symptomatic profile, it’s often the DSM approach. So, maybe having something a bit more holistic and a bit more solvent” – Participant 3, Group 2.*

Alongside difficulties with understanding mental health symptoms, two other notable areas were mentioned. Firstly, teaching students how to identify symptoms that are severe enough to warrant professional psychological support was highlighted. Many of the participants articulated difficulty in assessing their perceived need for mental health support.

*“the difficult part was thinking, convincing myself I need help. And that was it, it’s just getting over that first barrier and thankfully I did get over it. But the issue is that for me personally, that’s the biggest barrier for myself - realising I need help” – Participant 5, Group 1.*

“*we’re all at university, there’s a lot of other pressures going on, there’s a certain amount, everyone just expects you to be stressed, and there’s just certain expectations that you should be feeling that way. So, it’s difficult to then think to yourself okay, there’s a certain amount of this I should be feeling, but I’m now feeling too much” – Participant 6, Group 1.*

Other suggestions included: mental health interventions should explain when symptom awareness translates into seeking help, provide a checklist so students can cross-reference their symptoms, or include group discussions around mental health to facilitate self-reflection and greater awareness of symptoms.

*“I’d have very generic statements, ‘I am not enjoying what I used to enjoy’, ‘I feel like I’m tired all the time’, blaa blaa blaa. If you’re to say these out loud to certain individuals and ‘how many of these can you relate to?’, at this point it might trigger something to check themselves by” – Participant 5, Group 1.*

*“anyone who talks about their [mental health] issues and so forth publicly, the people in the audience will start to relate and then that will start triggering stuff and people will start talking about it, guaranteed every time” – Participant 5, Group 1.*

Secondly, participants suggested information about psychological treatment, namely the process, duration and general service structure would be helpful. Many participants acknowledged that they were unsure about which services were available, how to engage with them, and what kind of support they would receive if they did.

*“I think it’s a big thing about knowledge you need to know where to actually go, for instance I would normally, if I were to have mental health problems, I would normally think about the Student Union, just go maybe look at the Student Union but at the moment I have no idea where to look” – Participant 2, Group 3.*

*“we don’t actually talk about the process itself [i.e. therapy], how long does it take, what it looks like, when we should expect the first effect, why it’s not straight away, people don’t know this” – Participant 1, Group 1.*

The final point emerging from this theme highlighted logistical and structural barriers to seeking help. This included long waiting lists, lack of available support and slow administration surrounding university and professional mental health services.

*“when they’ve [i.e. a friend] looked for help the NHS has something like a 6-month waiting list, 6 months to see help. It’s a joke, it’s a joke” – Participant 5, Group 1.*

*“because of this high turnaround time I reckon that a lot of people might have the same feelings during exam time, during essay time, so a lot of people might want to talk to people and then it’s just going to get so convoluted everybody wants to talk and then I reckon services in this case might not be able to help people out*” *– Participant 2, Group 3.*

This theme summarises the help-seeking barriers identified by participants: difficulty identifying/understanding mental health symptoms, problems identifying whether support is actually needed, lack of clarity surrounding available services, how to engage with services, what support they would receive, long waiting lists and other structural barriers to treatment.

### Theme 5: strategies to sensitively engage male students

The most widely recommended method suggested during the focus groups to promote mental health in male students was paradoxically not to place emphasis on mental health or well-being. Indeed, this may overlap with a more informal approach advocated by some participants. Here, ‘mental health support’ was not perceived as beneficial and would result in a greater loss of time and resources if one were to attend. Having a title that does not reference mental health avoids this problem and was seen as less alienating, allowing for wider outreach to those who may not identify as having a mental health difficulty or who have symptoms that are not typically associated with mental health - such as problematic drinking, aggression and somatic symptoms.

*“well-being sort of seems to ‘ah it feels like I’m going to another session and I’m going to get lectured’ and it’s just a word I’ve heard a lot, it’s an empty wishy-washy word [i.e. well-being]” – Participant 7, Group 2.*

*“you know if you’re struggling with depression and what not as a man, let’s be real are you going to go to this workshop talking about men’s mental health? Probably not” – Participant 1, Group 2.*

Similarly, providing an incentive or clear short-term benefit would help tip this cost-benefit analysis more favourably.

*“So, I feel like if you have a side benefit to going to a workshop like that, that might be really cool” – Participant 5, Group 3.*

*“Something similar to this with some snacks, like with some food or something kind of… an incentive to come” – Participant 6, Group 1.*

Other recommendations included promoting interventions through student networks or clubs, pre-existing bodies within the university and face-to-face advertising, as opposed to university wide e-mails and posters, as it was considered more engaging resulting in potentially higher levels of attendance.

*“Getting societies involved, now I’m thinking about it, is a really really good idea ‘ cause you catch so many people like that, you catch the people at events, you catch a lot of different groups of people by getting societies involved” – Participant 5, Group 3.*

*“Yeah well, human contact, like ‘hey dude it’s actually quite cool come along’ and then you are much more inclined to go instead of seeing a poster” – Participant 5, Group 3.*

Finally, participants felt delivering mental health initiatives at the beginning of an academic year during orientation or ‘freshers’ week could elicit higher engagement. During this period, students have more time available to engage with extra-curricular activities and are more motivated to participate.

*“for freshers you just say ‘okay, now I have time’ you want to do stuff, you feel like you’ve got an obligation to actually do stuff, maybe like 3 weeks afterwards you’re like I don’t care anymore but at the start you want to do something, you want to be informed, and maybe that’s the best place to get to people so when they’re still motivated” – Participant 2, Group 3.*

In addition to this, delivering mental health initiatives during ‘critical’ or ‘darker’ months was also considered to be a good idea. Participants thought running interventions around exams and before the Christmas/winter break would be more appealing and relevant to male students.

*“then there should be like in these ‘dark months’ before exams” – Participant 4, Group 3.*

*“you introduce sessions maybe before Christmas and then before exams” – Participant 4, Group 3.*

This theme captures key strategies which might help attract male students to attend mental health initiatives, and more specifically seek help. Labelling the intervention as something other than mental health, providing a short-term incentive, advertising via pre-existing bodies and delivering initiatives during orientation and before exams were the most widely discussed strategies.

Overall, these five themes provide insight into how male students might think and how to better engage male students with mental health initiatives, possibly resulting in more effective and positive changes to psychological help-seeking.

## Discussion

These focus groups identify five themes relating to: protecting male vulnerability, providing a masculine narrative of help-seeking, differences over intervention format, difficulty knowing when and how to seek help and strategies to sensitively engage male students.

Engaging with mental health services was reported as threatening and intimidating for male students, which led to apprehension and reluctance to seek support. This supports previous findings, which suggest men and male students require more trusting relationships, assurance of confidentiality and good rapport when managing mental health difficulties [44–46]. The need for trust, confidentiality and good rapport may be due to components of stigma, characterised as one’s attitudes and misconceptions of mental health and those with a mental health condition [47, 48]. Stigmatising beliefs negatively impact help-seeking behaviours and attitudes which can account for the reluctance and apprehension experienced by the participants in this study [49–51]. Indeed, men often have greater stigmatising views of mental health compared to women [49, 52, 53]. By protecting the vulnerability male students experience with seeking help, it is likely to reduce the anticipated or experienced stigma. For example, building trust and emphasising confidentially can help dispel fears of being judged or personal information being shared outside the therapeutic setting. Along the same lines, providing social support within interventions may also reduce the emotional intensity and subsequent ‘threat-level’ of engaging. Social support can help encourage one to seek help, as being supported and validated by others helps to reduce one’s internalised stigma [54, 55]. Men often prefer group work [30] and have a greater propensity to seek help when there is positive social encouragement to do so [46]. Furthermore, male-only spaces that are gender-sensitive may help to validate men’s mental health concerns and guard against negative perceptions of help-seeking [56].

Participants discussed the notion of patriarchy, whereby the current world is seen as privileging, empowering and advantageous for men. In efforts to address this, society minimises male success/inequalities and magnifies female success/inequalities [57]. Subsequently, male students may discredit, invalidate or delegitimise their own concerns surrounding mental health and seeking professional support due to feelings of lack of entitlement, anticipated criticism or disapproval. These feelings may be heightened in the presence of female students, indicating a need for a male only-space.

The second theme related to seeking help which was characterised as dramatic, weak, less responsible, feminine, incompetent and less independent. Such stigmatising perceptions may contribute to greater self-criticism or self-stigma as these contradict traditional masculine stereotypes of strength, responsibility, self-sufficiency and control [58]. Indeed, evidence highlights that men are more likely to internalise stigmatising views held by the general public and that self-stigma mediates the relationship between masculine norms and help-seeking attitudes [15, 19, 50]. In the present study, a cost-benefit analysis emerged weighing up the advantages and disadvantages of seeking help in the context of a potential threat to one’s masculinity. Conversely, some students articulated help-seeking to be consistent with traditional masculine stereotypes. Framing help-seeking as a sign of strength, a display of responsibility or an act of self-growth could lead to more positive discourses surrounding mental health help-seeking and reduce the stigma associated with engaging. Indeed, this supports previous findings demonstrating male students who re-define help-seeking as a sign of strength adopt more positive help-seeking behaviours [19, 21].

Men who do seek help may feel inadequate or deviant from prescribed male norms [59]. Findings from these focus groups indicated that by normalising help-seeking and re-framing it to fit better within positive masculine norms, there is potential to improve service engagement, possibly through the reduction in self-stigma [16, 60]. Adjusting therapeutic environments to be male-specific, safe and male-friendly whilst adopting ‘male-positive’ values can assist with normalising help-seeking and reduce the stigma associated with seeking help [61]. Another way to achieve this is to incorporate male role-models into future work. This approach is often used within male help-seeking interventions [62], where evidence supports the use of celebrities to teach people about mental illness and is an effective strategy for reducing mental health stigma [63].

Although much of the current findings may align with previous literature regarding stigma and masculine norms, male students experience a broad range of barriers where factors such as stigma are not always the biggest obstacle [60]. The third theme reflected key differences amongst the participants. Consistently throughout, half the participants preferred an informal and fun setting for an intervention as this would be more interesting and enticing. Previously, the use of humour and funny mental health campaigns have been shown to increase awareness of mental health and promote greater interest in counselling services [64]. Furthermore, lay language and humour provides relational styles that are more familiar to men [65]. Indeed, this may help to explain why previous, more formal, interventions have struggled to engage men. In contrast, other participants expressed a preference for a formal setting. This was to help validate the significance of men’s mental health and allow for mental health concerns to be discussed in a safe and serious setting, similar to that of traditional therapies.

Another difference focused on the duration of the intervention. Some participants suggested shorter interventions may be preferable as they require less commitment. This is corroborated by other discourses identified from these focus groups, where many of the students appear to undergo a cost-benefit analysis when deciding whether to seek help. In this instance, a brief intervention reduces the associated costs of engaging with support. Conversely, those expressing the need for a serious and formal setting were of the view that a prolonged and frequent intervention was required, due to the pervasiveness of mental health difficulties. One way of reconciling these discrepancies would be to blend both approaches, as this may appeal to more male students [61, 66]. Alternatively, a ‘one-size-fits-all’ approach is unlikely to solve the current issues and a variety of different intervention formats could be assessed to see which is more appropriate for male students. Certainly, the development of brief and informal interventions requires testing, as this approach is not currently provided by traditional mental health services.

The fourth theme captured the difficulties male students have with identifying mental health symptoms and knowing whether and when it is appropriate to seek support. Male students appear to have greater difficulty in identifying mental health symptoms compared to female students [67, 68]. Improving mental health literacy is not a novel finding and has been a key target area for previous student mental health interventions [69–71]. The rationale underpinning mental health literacy programmes serves to target mental health knowledge by improving one’s ability to recognise mental health symptoms, have sufficient knowledge of treatment, and appropriate self-help strategies to facilitate help-seeking [72]. Similarly, mental health literacy interventions and campaigns can assist with improving mental health awareness whilst reducing stigmatising perceptions of mental health [73, 74]. Positive improvements in both these domains can elicit greater help-seeking. The current findings extend this rationale further by highlighting the difficulty male students in particular have with relating symptoms to seeking support. To overcome this, participants recommended providing more concrete means to self-evaluate their symptoms, such as checklists and group discussions. Improving symptom knowledge and providing more specific clinical thresholds can help facilitate earlier detection and intervention of mental health difficulties.

Likewise, many students were unsure about how to access mental health support. Indeed, positive changes to help-seeking have been seen when services are sign-posted [62]. Furthermore, male students were uncertain about what actually happened during therapy. This confirms previous research, whereby men often fail to understand various treatment options (particularly psychological therapies) and are unaware of the positive elements of help-seeking and how it relates to recovery [75]. It is clear from these findings that male students require information about how treatment works, it’s content and duration and what progress may look like. Additional barriers students mentioned when seeking support included the logistical and structural barriers to services. Although dependent on funding, services should seek to make self-referrals less cumbersome and increase the availability of support staff to reduce waiting times for all students.

Finally, the fifth theme highlighted another issue when promoting mental health initiatives, as participants reported labels of ‘mental health’ or ‘well-being’ should be avoided as they could discourage attendance. These labels can be alienating and are perceived as being less benign than terms not related to mental health and they are likely to elicit stigmatising beliefs and negative perceptions of mental health. Men often reject services that use ‘psychiatric’ or ‘diagnostic’ frameworks that seek to label emotional distress as a mental illness [76]. Avoiding a name that emphasises mental health could also help to engage male students who do not identify as having a formal mental health diagnosis but may be experiencing distress. Providing a secondary incentive was also recommended to help shift perceptions of help-seeking towards being a more positive and worth-while activity.

These focus groups also advised promoting mental health initiatives through pre-existing social networks such as university societies. This is a preferred method of communication for young adult males (18–25 years), as they are more likely to seek in-person mental health services when encouraged by their family or partner, whilst peer support increases in-person mental health service use after adolescence [77].

Lastly, delivering mental health initiatives during university orientation week(s) and preceding exam periods was recommended. Previous research supports this, as lack of time is a frequent barrier students face when engaging with mental health support and thus it may be more acceptable to position mental health initiatives when students have more time resources available or within close proximity such as student unions and halls of residences [78–80]. Alternatively, it was proposed that mental health initiatives should be delivered during exam periods, as they can cause or contribute to higher levels of emotional distress [81]. Although engaging before exams would be more time-costly, mental health support was perceived as having a greater benefit at this time. Mental health initiatives for male students should be positioned when it is most likely to engage them, particularly at the beginning of university (i.e. orientation week/freshers) and during exam periods.

Overall, it is hoped that these findings can be used alongside other recommendations [56, 62, 65] to design more effective mental health interventions for male students to improve both their uptake and engagement.

### Strengths & Limitations

Valuable insights have been gained from male students regarding the design and development of mental health initiatives for this population. A strength of this investigation included the use of two independent reviewers throughout thematic coding to reach consensus. Additionally, this investigation consulted the YPMHAG resulting in a more tailored and appropriate topic guide for this study. This may help explain why retention was relatively high (75%), with only 16 students (25%) losing contact after approaching the research team.

Furthermore, the current investigation purposely included participants who have (50%) and have not (42%) previously sought help for mental health. In turn, this enabled a broader overview of experiences male students may face when seeking help.

This study is not without limitations. Although the identified themes were sent to participants for data validation, none of them responded, which makes it difficult to state with certainty that participants felt the finalised themes capture their responses. Additionally, the current investigation was conducted by a novice researcher with each focus group facilitated by male students. This may have methodological implications, particularly the possibility of influencing the focus group discussions with their own biases. However, the researcher took part in several training courses prior to the study, regarding how to facilitate focus groups and conduct thematic analysis. Additionally, the research was supervised by experienced qualitative researchers throughout all stages of the study, which should have mitigated this risk.

Lastly, thematic analysis was chosen to identify the key patterns and themes that emerge from the data [34]. As part of this process, ‘data reduction’ occurs to condense and synthesise the most prominent ideas within the data [82]. This could mean more nuanced ideas and recommendations are lost due to not aligning with a broader, reoccurring theme. This may be of importance for different sub-groups of male students, as they may be faced with subtle differences when seeking help for mental health difficulties.

### Reflexivity

It is important to acknowledge two key aspects that may have influenced the results presented in this paper. In this study, only male students were recruited and the researchers facilitating the focus groups (ISO and VT) were both male. This may have allowed male students to feel more comfortable when talking about mental health help-seeking. This was particularly apparent when students discussed masculine stereotypes and the notion that having a male only space was of importance. Similarly, both the focus group facilitators were students currently studying at the same institution as the participants. The researchers kept a reflexive diary and felt they had greater contextual understanding of the discourses provided, allowing for greater rapport and freedom for participants to express their thoughts. It is possible that as a result, the current focus groups provide a more detailed and accurate account of male students’ experience of help-seeking.

## Conclusion

Student mental health and poor male help-seeking is a major concern and providing the right response is currently being debated. The current investigation provides a detailed account of suggestions from current students about how to improve mental health initiatives for male students. It is hoped that the themes of protecting male vulnerability, providing a masculine narrative of help-seeking, differences over intervention format, difficulty knowing when and how to seek help and strategies to sensitively engage male students can be considered and implemented when designing future mental health interventions that seek to improve male students’ overall well-being or willingness to seek psychological support.

## Supplementary information

**Additional file 1 Supplementary Material 1.** Focus Group Topic Guide (including supportive questions)

## Data Availability

As this is a qualitative design, a data set is not available. The transcripts analysed in this study are not publicly available due to participants not consenting to have this information shared with a third party.

## Abbreviations

KCL: King’s College London
UK: United Kingdom
YPMHAG: Young Person’s mental health advisory group
