# Supplementary material for: Engaging male students with mental health support: a qualitative focus group study
Source: BMC Public Health. 2020 Jul 24;20:1159. doi: 10.1186/s12889-020-09269-1 (PMC7379819; doi:10.1186/s12889-020-09269-1)
Supplement: Supplementary file 1 — Additional file 1 Supplementary Material 1. Focus Group Topic Guide (including supportive questions) [file 12889_2020_9269_MOESM1_ESM.docx]

**Supplementary Material**

Supplementary Material 1: Focus Group Topic Guide (including supportive questions)

Q1) Why don’t student’s seek help for mental health?

Q2) Why don’t male students seek help for mental health?

2a) What would your thoughts/feelings be about opening/asking for help?

2b) If you were finding it hard would you know where to go/who to ask for help?

2c) How able are you at working out how you’re feeling?

2d) Are there any social/cultural pressures that would discourage you?

Q3) What would encourage you to seek mental health help if you had a problem?

3a) Would celebrities or other men with difficulties change your perception?

3b) Would you like to receive information about diagnosis and treatment?

3c) Would you like to know how to recognise specific symptoms?

3d) Would you prefer to complete tasks and learn new skills (as opposed to talking)?

3c) Would you like to receive information about where to access support?

3d) Would it help if help-seeking was explained/reframed as a sign of strength?

Q4) Is there anything that should not be included/would put you off?

Q5) If we were to offer something, how would you like it to be?

5a) The possibility of male only spaces/ men only groups.

5b) Would you prefer a brochure or a group workshop?

5c) If a workshop, how many sessions, how long for?

5d) Would you prefer to take part in problem solving activities?

Q6) What is a good way to market the intervention to men?

6a) Should we make it clear it’s about mental health or not?

6b) What is the best way to inform people about this intervention?

6c) What should the intervention be called?

6d) Where should this take place?

6e) Does there need to be incentives for coming, if so what?

6f) Should we use social media? If so, which platform, level of engagement?

Q7) Is there anything else you would like to add?
